# Supplementary material for: Association between the Potential Influence of a Lifestyle Intervention in Older Individuals with Excess Weight and Metabolic Syndrome on Untreated Household Cohabitants and Their Family Support: The PREDIMED-Plus Study
Source: Nutrients. 2020 Jul 3;12(7):1975. doi: 10.3390/nu12071975 (PMC7400558; doi:10.3390/nu12071975)
Supplement: Supplementary file 1 [file nutrients-12-01975-s001.pdf]

Supplementary material

General characteristics of the household PREDIMED-Plus sons and daughters according to Mediterranean Diet adherence categories.

	Adherence to the MedDiet of the PREDIMED-Plus participant			
	≤7<br>n=9	8 - 10<br>n=28	>10<br>n=51	*p*-overall
**PREDIMED-Plus lifestyle intervention**				
Sons and daughters adherence to the MedDiet (score)	5.56 (2.92)	6.71 (2.07)	7.25 (2.25)	0.107
Sons and daughters physical activity (METs/min/week)	2670 (1602)	2976 (2474)	2319 (1991)	0.445
	**Sons and daughters adherence to the MedDiet (score)**			
	**≤7<br>n=55**	**8 - 10<br>n=20**	**>10[c]<br>n=13**	***p*-overall**
**Sociodemographic characteristics**				
**Age (years)**	31.8 (8.40)	31.3 (7.44)	36.9 (7.86)	0.100
**Sex***				
Women	30.9 (17)	40.0 (8)	23.1 (3)	0.602
Men	69.1 (38)	60.0 (12)	76.9 (10)	
**Education level***				
University	9 (16.4 (9)	10.0 (2)	53.8 (7)	0.078
High-school	18.2 (10)	35.0 (7)	15.4 (2)	
Secondary-school	29.1 (16)	25.0 (5)	7.69 (1)	
Elementary-school	36.4 (20)	30.0 (6)	23.1 (3)	
**Chronic disease prevalence***				
Hypertension	31.5 (17)	20.0 (4)	38.5 (5)	0.531
Dyslipidemia	29.1 (16)	15.0 (3)	23.1 (3)	0.526
Type 2 diabetes mellitus	5.45 (3)	15.0 (3)	15.4 (2)	0.256
**Anthropometric measures**				
Weight (kg)	80.8 (16.4)	72.2 (10.5)	77.7 (10.7)	0.187
Height (cm)	172 (8.69)	171 (8.79)	175 (11.1)	0.580
BMI (kg/m^2)	26.9 (5.45)	24.2 (3.75)	25.9 (4.73)	0.242
**Lifestyle**				
Physical activity (METs/min/week)	1659 [1051; 2979]	2288 [901; 4041]	2965 [1929; 4147]	0.281
Eating together (times per week)	8.00 [5.00; 14.0]	7.50 [5.75; 10.2]	9.00 [7.00; 14.0]	0.403
**Social characteristics of the household PREDIMED-Plus cohabitants**				
Family function (score)	8.00 [8.00; 10.0]	9.00 [8.00; 10.0]	9.00 [9.00; 9.00]	0.484
Family APGAR items				
Adaptability*				0.822
Hardly ever	0.0 (0)	0.0 (0)	0.0 (0)	
Some of the time	13.0 (3)	8.33 (1)	20.0 (1)	
Almost always	87.0 (20)	91.7 (11)	80.0 (4)	
Partnership*				0.722

Hardly ever	0.0 (0)	0.0 (0)	0.0 (0)	
Some of the time	43.5 (10)	33.3 (4)	20.0 (1)	
Almost always	56.5 (13)	66.7 (8)	80.0 (4)	
Growth*				0.980
Hardly ever	4.3 (1)	0.0 (0)	20.0 (1)	
Some of the time	34.8 (8)	41.7 (5)	20.0 (1)	
Almost always	60.9 (14)	58.3 (7)	60.0 (3)	
Affection*				0.073
Hardly ever	0.0 (0)	0.0 (0)	0.0 (0)	
Some of the time	39.1 (9)	8.33 (1)	60.0 (3)	
Almost always	60.9 (14)	91.7 (11)	40.0 (2)	
Resolve*				-
Hardly ever	0.0 (0)	0.0 (0)	0.0 (0)	
Some of the time	0.0 (0)	0.0 (0)	0.0 (0)	
Almost always	100.0 (23)	100.0 (12)	100.0 (5)	
Social support (score)	48.0 [41.0; 51.0]	48.0 [47.0; 54.2]	49.0 [47.0; 51.0]	0.375
Social support sub-scales				
Affective support (score)	18.0 [15.0; 19.0]	18.0 [17.0; 20.0]	18.0 [16.0; 18.0]	0.528
Confidential support (score)	29.0 [26.0; 32.0]	31.5 [29.8; 34.2]	31.0 [31.0; 33.0]	0.182

Data are presented as mean (SD) and as median [IR] for continuous variables, and as % (n) for categorical variables*

General characteristics of the household PREDIMED-Plus sons and daughters according to physical activity categories.

	Physical activity of the PREDIMED-Plus participant		
	**Very Active n=60**	**Active n=28**	***p*-value**
Sons and daughters adherence to the MedDiet (score)	6.93 (2.34)	6.86 (2.27)	0.885
Sons and daughters physical activity (METs/min/week)	2496 (2033)	2697 (2362)	0.713
	**Partner physical activity**		
	**Very Active n=39**	**Active n=43**	***p*-value**
**Sociodemographic characteristics**			
**Age (years)**	32.6 (8.72)	31.7 (7.95)	0.629
**Sex***			
Women	25.6 (10)	41.9 (18)	0.189
Men	74.4 (29)	58.1 (25)	
**Education level***			
University	15.4 (6)	23.3 (10)	0.778
High-school	25.6 (10)	20.9 (9)	
Secondary-school	23.1 (9)	25.6 (11)	
Elementary-school	35.9 (14)	30.2 (13)	
**Chronic disease prevalence***			
Hypertension	28.9 (11)	32.6 (14)	0.912
Dyslipidemia	17.9 (7)	27.9 (12)	0.421
Type 2 diabetes mellitus	15.4 (6)	4.6 (2)	0.142
**Anthropometric measures**			
Weight (kg)	75.6 (13.1)	80.6 (15.5)	0.189
Height (cm)	173 (8.88)	171 (9.20)	0.211
BMI (kg/m^2)	24.8 (3.72)	27.4 (5.84)	0.048
**Lifestyle**			
Adherence to the MedDiet (score)	7.41 (2.46)	6.81 (1.95)	0.231
Eating together (times per week)	9.00 [7.00; 14.0]	7.00 [6.00; 12.5]	0.443
**Social characteristics of the household PREDIMED-Plus cohabitants**			
Family function (score)	9.00 [8.00; 10.0]	8.50 [8.00; 10.0]	0.666
Family APGAR items			
Adaptability*			1.000
Hardly ever	0.0 (0)	0.0 (0)	
Some of the time	14.3 (3)	11.1 (2)	
Almost always	85.7 (18)	88.9 (16)	
Partnership*			0.979
Hardly ever	0.0 (0)	0.0 (0)	
Some of the time	33.3 (7)	38.9 (7)	

Almost always	66.7 (14)	61.1 (11)	
Growth*			0.439
Hardly ever	0.0 (0)	2 (11.1%) (2)	
Some of the time	38.1 (8)	6 (33.3%) (6)	
Almost always	61.9 (13)	10 (55.6%) (10)	
Affection*			0.733
Hardly ever	0.0 (0)	0.0 (0)	
Some of the time	28.6 (6)	38.9 (7)	
Almost always	71.4 (15)	61.1 (11)	
Resolve*			-
Hardly ever	0.0 (0)	0.0 (0)	
Some of the time	0.0 (0)	0.0 (0)	
Almost always	100.0 (21)	100.0 (18)	
Social support (score)	49.0 [45.5; 53.0]	47.0 [42.5; 49.8]	0.110
Social support sub-scales			
Affective support (score)	18.0 [17.0; 19.5]	17.0 [15.2; 18.8]	0.143
Confidential support (score)	32.0 [28.5; 34.0]	29.5 [28.0; 32.8]	0.261

Data are presented as mean (SD) and as median [IR] for continuous variables, and as % (n) for categorical variables*
